# Novel bilateral symmetrical congenital transverse upper and lower limb deficiencies in siblings in Ethiopia: two case reports

**DOI:** 10.1186/s13256-022-03418-3

**Published:** 2022-06-02

**Authors:** Amen Samuel Melaku, Fiker Tadesse Bekele, Yilkal Muchie Dires, Laurence Wicks

**Affiliations:** 1 Mckinsey and company management consulting, Doha, Qatar; 2 All Africa TB Leprosy and rehabilitation training (ALERT) Center, Addis Ababa, Ethiopia; 3 CURE Ethiopia Children’s Hospital, Addis Ababa, Ethiopia; 4 Center for Innovative Drug Development and Therapeutic Trials for Africa (CDT-Africa), College of Health Sciences, Addis Ababa University, P.O. Box 9086, Addis Ababa, Ethiopia

**Keywords:** Bilateral limb deficiency, Siblings, Genetic link, Ethiopia

## Abstract

**Background:**

Transverse congenital limb deficiency is a common limb deficiency where there is normal limb development until a certain point, beyond which no anatomical structure exists. Typically, this presents as an isolated and spontaneous abnormality as a result of arrest during limb bud development. Transverse bilateral deficiency in both upper and lower limbs is not well described.

**Case presentation:**

We report the cases of two female Ethiopian amhara siblings, aged 6 years and 5 months, respectively, from Ethiopia with similar transverse bilateral upper and lower limb deficiencies. The sisters were born from the same parents and have similar phenotypic presentations. Neither of them have other syndromic features or systemic manifestations. The siblings are currently on follow-up and are receiving assistance by specialist orthotists, who are working to improve walking and also providing adaptive equipment to facilitate self-care and feeding.

**Conclusion:**

The relationship of the patients and the similarity of phenotypical presentations suggests a strong genetic link.

## Background

Congenital limb deficiencies occur with an incidence of 5–9.7 per 10,000 live births [1]. These disorders are characterized by aplasia or hypoplasia of one or more bones of the limbs. After decades of disagreement on nomenclature, in 1989 an international standard nomenclature was adopted [2]. This nomenclature classifies congenital limb deficiencies into two broad categories: transverse and longitudinal.

Transverse congenital limb deficiencies are limb disorders where there is normal development until a certain point, beyond which no tissue exists [3]. These disorders can be isolated or associated with other congenital anomalies, often occurring around the same time as limb bud development during weeks 4–8 of gestation. Isolated transverse limb deficiency is the most common congenital limb deficiency, occurring in 3 out of every 10,000 births, and is considered a sporadic occurrence as a disruption to normal limb bud development [4]. The risk of similar abnormalities in future pregnancies is therefore not considered greater than the risk in the general population.

We present a case report of two siblings who have transverse bilateral upper and lower limb deficiencies. The sisters were born from the same parents and have similar phenotypic presentations. Whilst environmental etiologies cannot be excluded, the similarity of phenotypic presentations in these sisters suggests an underlying genetic cause.

## Case presentations

### Case 1

This 6-year-old Ethiopian amhara female presented with symmetrical distal transverse deficiencies of both upper and lower limbs. She has no other syndromic features or systemic manifestations.

The patient is capable of walking with the support of orthotics. She has adapted to use her forearm for several activities including, but not limited to, eating and writing. However, speed and certain activities that require fine motion are difficult to achieve (Fig. 4).

#### Case history

This patient was first presented after delivery of her younger sister (case 2). Medical records about antenatal follow-up or delivery are not available. Developmental milestones have been met at the appropriate age. Further, examination of the respiratory system, cardiovascular system, and abdomen was normal.

#### Physical examination results

- In the upper limbs, the patient has normal shoulder, elbow, and wrist function with transverse deficiency distal to carpal bones.
- The child has a normal spine.
- In the lower limbs, the patient has normal hip, knee, and ankle function with transverse deficiency distal to the hind foot (Fig. 5).

#### Results of other investigations

- Abdominal ultrasound and echocardiograph are unremarkable.
- See Fig. 6 for X-ray images of case 1.

#### Management plan

The child has been reviewed in a pediatric orthopedic hospital. No surgical intervention is planned. While some surgical procedures are described for children who have suffered loss of hands, such as the Krukenberg procedure [5], it is felt that this will not afford this patient great additional function benefit but may be socially stigmatizing. Assistance is being received from specialist orthotists, who are working to improve walking and also providing adaptive equipment to facilitate self-care and feeding.

### Case 2

**Fig. 1:**
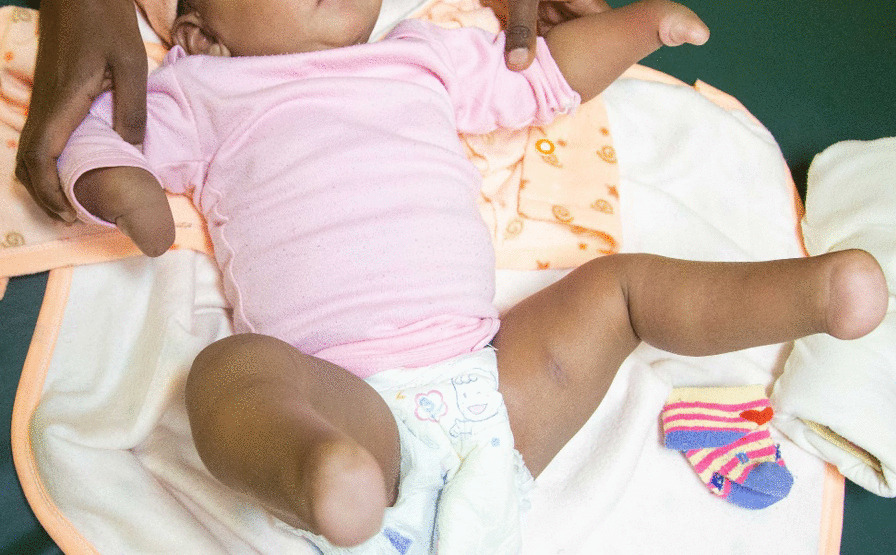
Case 2, a 5-month-old female with bilateral absence of hands and feet

This patient is a 5-month-old Ethiopian amhara female, born to the same parents as case 1. She presents with a similar clinical feature of symmetrical distal transverse deficiency of both upper and lower limbs (Fig. 1, patient 2, 5-month-old female with bilateral absence of hands and feet).

#### Case history

This child was monitored through antenatal follow-up until delivery at 39 weeks’ gestation.

Delivery was by spontaneous vaginal delivery, and birth weight was 2.5 kg.

The mother is 26 years old. There was no medical or obstetric complication during pregnancy or delivery. There was no history of trauma to abdomen or radiation exposure during antenatal period, and the mother was neither a smoker nor an alcoholic.

After the delivery, the patient was brought into the neonatal intensive care unit (NICU) to be evaluated for any potential congenital anomaly associated with the absence of hands and feet.

#### Physical examination

Clinical findings were similar to her older sister, with normal limbs other than transverse deficiency distal to the carpal bones and hindfeet. Examination of the respiratory system, cardiovascular system, and abdomen was unremarkable.

#### Other investigations

Ultrasound examination during pregnancy did not reveal any anomaly. Postnatal examination of the placenta was normal, and histopathology did not reveal any significant findings. Ultrasound examination at birth of abdomen and cranium was normal.

At 5 months of age, ultrasound of the abdomen was unremarkable. Echocardiography demonstrated a 1.2-mm patent ductus arteriosus (PDA) shunting left to right. Otherwise, all four valves appeared structurally normal with left ventricular ejection fraction of 72% confluent and good-sized branch pulmonary arteries, and there is no coarctation.

#### Management plan

This child has also been reviewed at a specialist pediatric orthopedic hospital. No surgical intervention is planned. When she begins to stand, the orthotists will assist to improve balance and facilitate walking.

**Fig. 2:**
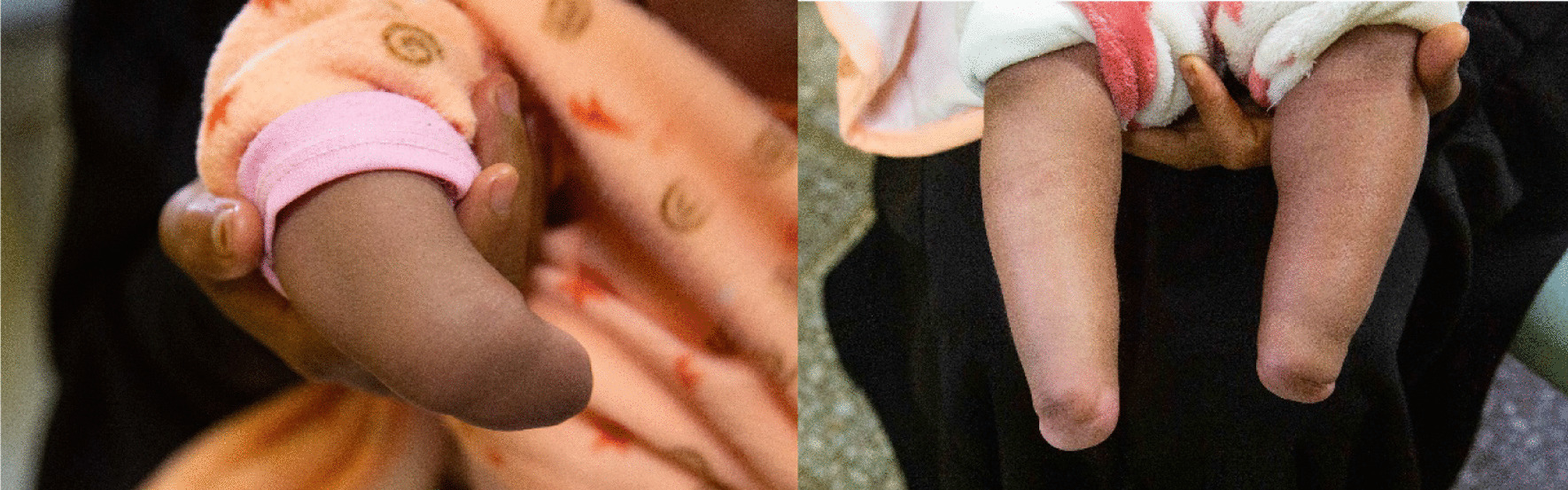
Images of upper (left) and lower (right) limb of case 2

**Fig. 3:**
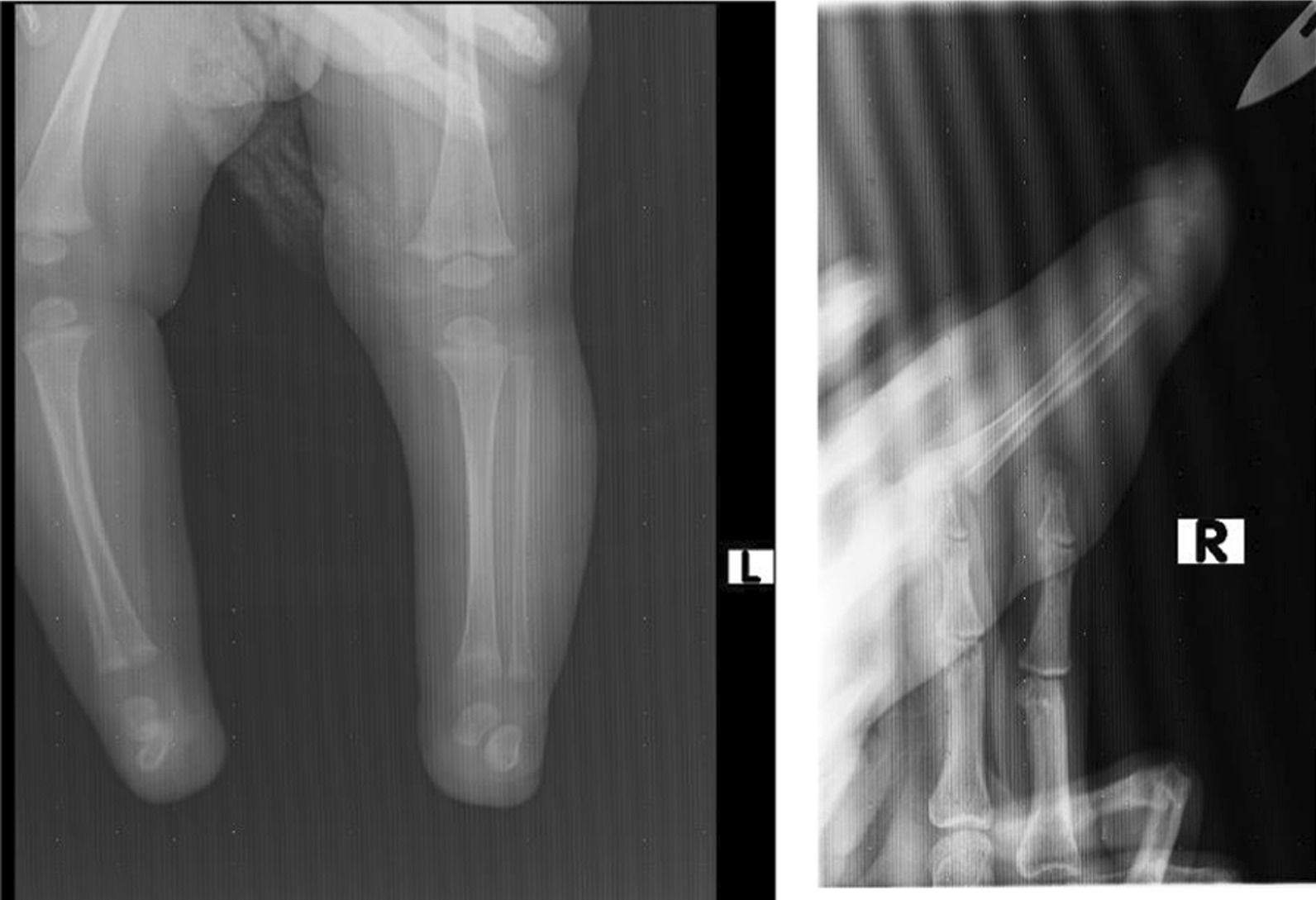
X-ray images of upper (right) and lower (left) limbs for case 2

**Fig. 4:**
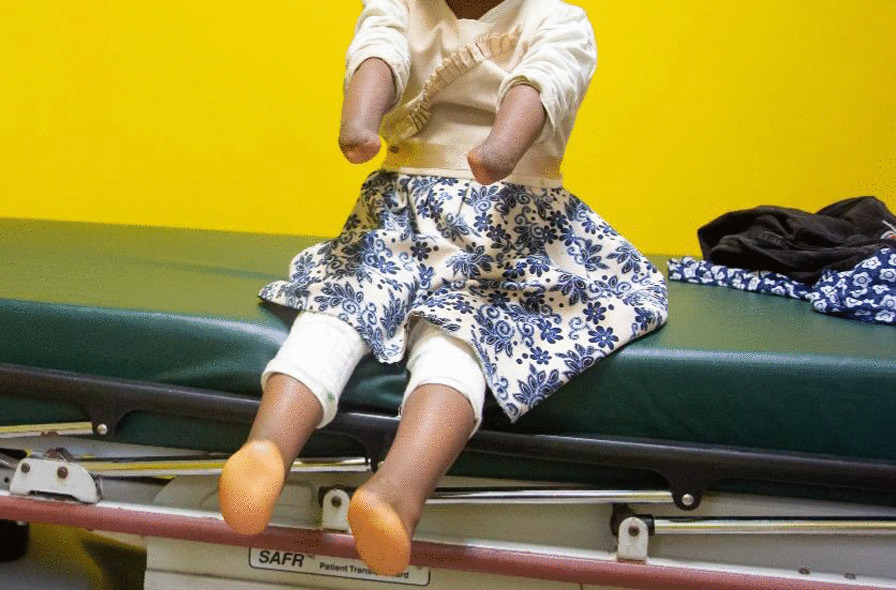
Case 1: 6-year-old female with bilateral deficiency of hands and feet

**Fig. 5:**
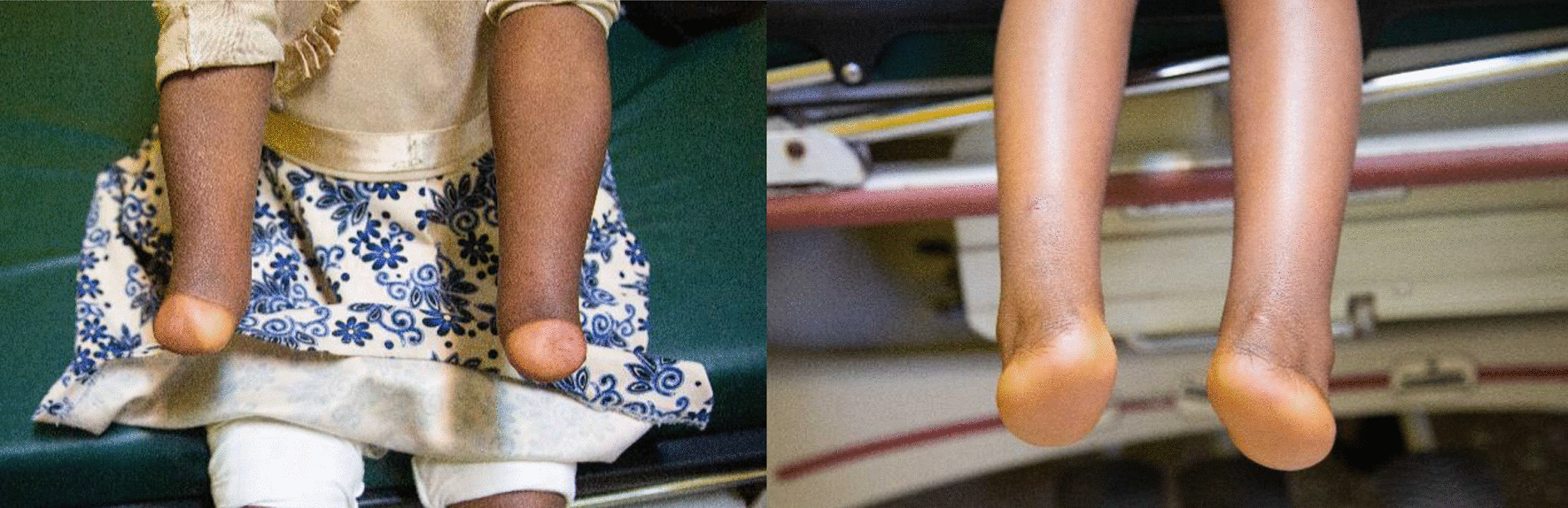
Images of upper (left) and lower (right) limb of case 1

**Fig. 6:**
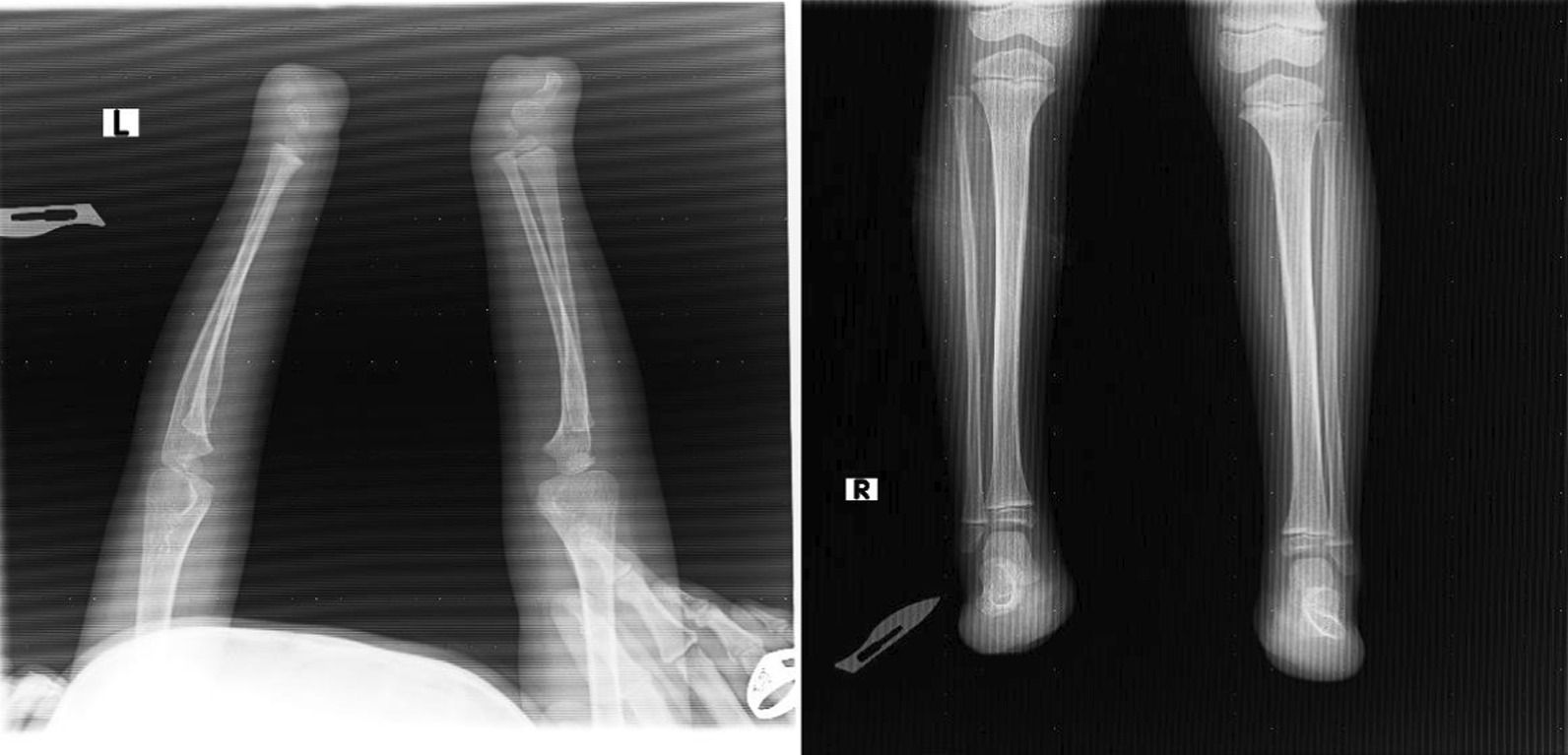
X-ray images for case 1

Regarding the cardiac abnormality, it is expected to close spontaneously without any long-term consequences (Figs. 2, 3, 4, 5, 6).

### Other details

These sisters are the only children born to these parents. However, the couple recently conceived a third child, who was terminated at 18 weeks 4 days after intrauterine ultrasound indicated the presence of similar abnormalities.

**Fig. 7:**
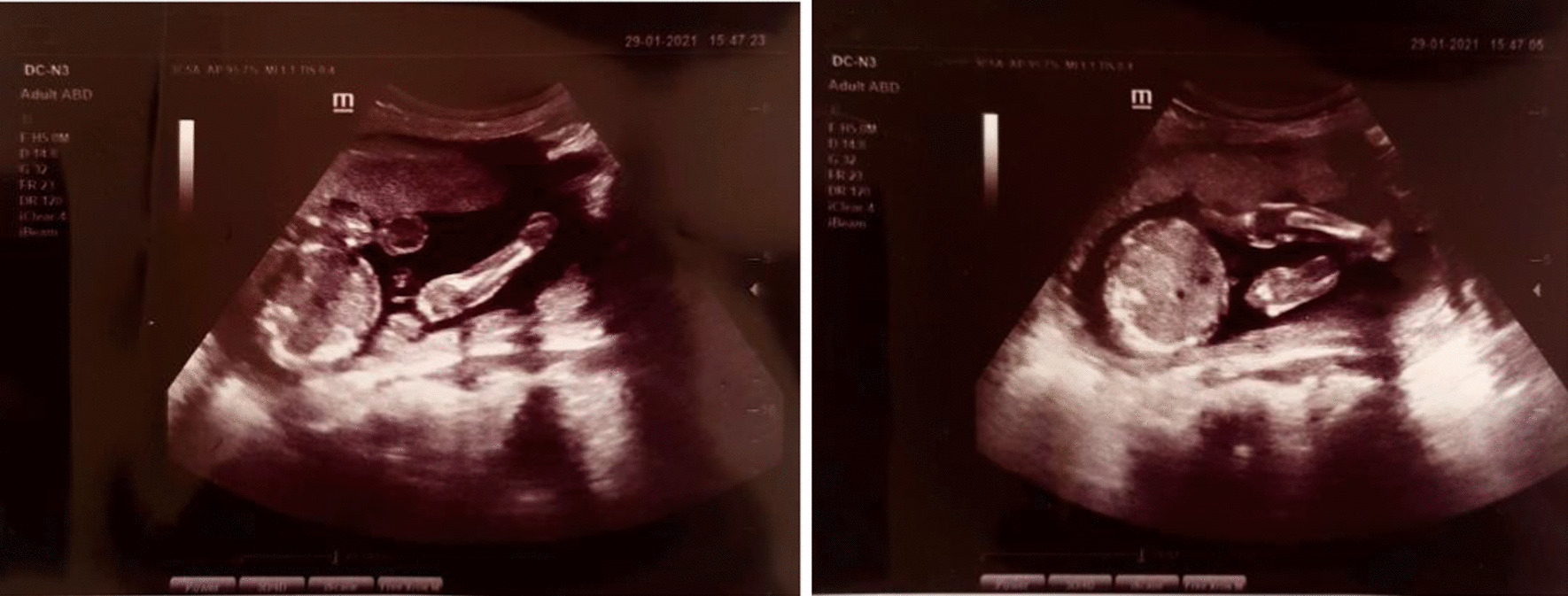
Ultrasound images of second-trimester intrauterine pregnancy at 18 weeks 3 days, which was later terminated

See Fig. 7 for ultrasound images of second-trimester intrauterine pregnancy at 18 weeks 3 days.

In addition, the father has two sons from a previous marriage, neither of whom have any known abnormalities.

The parents are unaware of any other family members affected in the same way. They are unwilling to disclose any consanguinity.

## Discussion

Archeiropoda (Gr. a = absence; cheir, cheiros = hand; pous, podos = foot) is a rare disorder that describes the absence of the distal extremities. Most individuals with the condition have bilateral deficiency beyond the distal epiphysis of the humerus and distal portion of the tibial diaphysis [6]. Rarely, some individuals present with an ectopic bone at the distal end of the humerus [7].

Archeiropodia has an autosomal recessive inheritance pattern. It was first described in 1929 in Brazil, with half of a family of 12 children affected [8, 9]. Studies estimate that the prevalence of this condition in Brazil is approximately 1 in 250,000 births [10, 11]. Genetic analysis of five families with archeiropodia revealed a common mutation in *C7orf2*, the human ortholog of the mouse *Lmbr1* gene [11]. This mutation was observed at both genomic DNA and messenger RNA (mRNA) levels [11]. This mutation leads to a premature stop codon, producing a transcript lacking exon 4. In heterozygotes, this mutation does not result in phenotypic manifestations of limb disorder. This condition has only been described in families originating from South America, with cases outside Brazil reported in Argentina and Puerto Rico [9].

The sisters described in this case have different phenotypical presentations than described in literature. In addition, these siblings do not have associated systemic disorders and are meeting proper developmental milestones. As such a disorder is novel, it is important to conduct further investigations to identify which genes are involved, as this could enhance understanding of the molecular mechanism of limb development in humans. Further genetic analysis of the family might elucidate how this condition emerged in the continent of Africa. However, due to local laboratory unavailability, we have not carried out gene sequencing of the siblings for chromosomal analysis.

## Conclusion

These sisters with the same biological parents present with the absence of hands and feet without any systematic manifestations. Even though our initial diagnosis for these patients was archeiropodia, their phenotypic manifestations are significantly different from all other patients with the condition. This suggests that the genetic cause of their condition could also be different. Therefore, we suspect that this could be a very rare genetic disorder that has not yet been described in the world. Thus, it is important to conduct further research on this family to identify the genetic cause of the disease, which could also enhance knowledge of limb development genes.

## Data Availability

Data used for all analyses are included in the manuscript.
